# Mechanism-resolving development of solid-tumor CAR T-cell therapy: linking programmable control to tissue pharmacodynamics

**DOI:** 10.3389/fimmu.2026.1983828

**Published:** 2026-10-22

**Authors:** Manh-Cuong Vo, Sang Ki Kim, Woo Kyun Bae, Nodirjon Ruzimurodov, Sung-Hoon Jung, Je-Jung Lee

**Affiliations:** 1 Institute of Research and Development, Duy Tan University, Danang, Vietnam; 2 Research Center for Cancer Immunotherapy, Chonnam National University Hwasun Hospital, Hwasun, Republic of Korea; 3 Vaxcell-Bio Therapeutics, Hwasun, Republic of Korea; 4 Department of Hematology-Oncology, Chonnam National University Hwasun Hospital and Chonnam National University Medical School, Hwasun, Republic of Korea; 5 Institute of Immunology and Human Genomics of the Academy of Sciences, Tashkent, Uzbekistan

**Keywords:** adaptive trial design, CAR T-cell therapy, mechanism attribution, solid tumors, tissue pharmacodynamics

## Abstract

Solid-tumor CAR T-cell programs increasingly combine biomarker selection, lymphodepletion, manufacturing-state optimization, regional or repeated administration, and engineered control modules. This growing complexity creates a distinct translational problem: the same response, non-response, or toxicity can remain compatible with several competing explanations, obscuring which component actually operated in patient tissue. Mechanism-resolving development addresses this problem by treating each consequential engineering claim as a prospectively testable proposition embedded within the product-trial system. Four questions must be resolved: which patient-relevant state was present, which control action altered it, which proximal tissue transition followed, and which unsafe or limiting state emerged next. Each claim should therefore be paired with a clinically credible lower-burden counterfactual, mechanism-proximal tissue pharmacodynamics, a prespecified falsification criterion, a rescue strategy, and an adaptive action. Boolean coincidence and veto circuits, temporal IF-THEN systems, quantitative antigen-density filters, and genotype-conditioned activator-blocker designs require distinct representations, transition-state assays, and tissue biomarkers. Clinical evidence from CLDN18.2-directed satri-cel, compartmental central nervous system programs, hypoxia-responsive CEA therapy, GPC3-armored products, PSMA programs, and early HLA-loss-gated Tmod therapy illustrates that clinical maturity and mechanistic attribution are not equivalent. Programmability becomes translationally meaningful when the operation of an added control can be distinguished from credible alternatives in patient-relevant tissue, enabling complex cellular engineering to generate falsifiable, cumulative, and clinically actionable knowledge.

## Highlights

- Clinical outcome alone cannot identify which component of a multi-component CAR T-cell program produced benefit or harm.
- Mechanism-resolving development must identify the relevant state, the control action, the proximal tissue transition and the failure state that follows.
- Every consequential module should be tested against a clinically credible lower-burden intervention aimed at the same biological limitation.
- Clinical maturity, mechanism-attribution strength and evidence direction are independent; negative outcomes can define highly informative safety or escape boundaries.
- Boolean, temporal, quantitative and activator-veto architectures require different transition-state assays and tissue biomarkers.
- The preferred architecture is non-dominated across expected benefit, safety, observability, reversibility, manufacturing and clinical-operational burden.

## 1 Introduction

### 1.1 From engineering abundance to mechanism resolution

CAR T-cell therapy demonstrated that a living immune cell can be manufactured as a medicine, expand after administration and generate durable antitumor effects. The first approved products benefited from accessible disease, broadly exposed lineage antigens and clinically manageable depletion of normal B-lineage compartments. Solid tumors rarely reproduce this alignment. Antigen density and spatial coverage, organ reserve, abnormal vasculature, matrix architecture, nutrient competition, suppressive myeloid states and the differentiation state of the infused T cell jointly determine therapeutic index (1–7).

Target selection, receptor architecture, trafficking, cellular fitness, tumor-microenvironmental resistance, regional delivery and external control have matured as distinct translational domains (5–15). Their increasing combination within a single program creates a different problem: the same clinical outcome may remain compatible with several explanations. Added capability can therefore increase uncertainty about which mechanism actually operated unless measurement and comparison are designed with the product.

The unresolved problem is epistemic as well as biological. In a multi-component product-trial system, response does not establish which component caused benefit, and non-response does not reveal which component failed. A biomarker-selected patient may receive lymphodepletion, a newly manufactured cell state, an engineered receptor, repeated administration and a regional route in the same program. Peripheral CAR-T expansion, radiographic response and toxicity alone cannot distinguish target biology, exposure, cell state, circuit performance or ecosystem change. This limitation is evident in early multicomponent clinical programs. In BNT211-01, peripheral CAR-T kinetics and radiographic outcomes were informative, but small heterogeneous cohorts and variable CARVac timing precluded determining the contribution of vaccine-mediated amplification to antitumor activity (16). Similarly, hypoxia-responsive CEA CAR-T therapy linked peripheral expansion to dose, route and response, while available compartmental measurements did not directly establish tumor-selective hypoxia-gated CAR activation, leaving route and circuit effects partially confounded (17). Conversely, locoregional IL-13Rα2 CNS therapy and PSMA-directed TGFβ-resistant CAR-T therapy incorporated CSF or paired-tissue pharmacodynamics, illustrating how measurements in the disease compartment can strengthen—without by themselves proving—component-level attribution (18, 19). Here, tissue pharmacodynamics refers to mechanism-proximal measurements in the compartment in which the control claim is expected to operate, rather than to a universal requirement for tumor biopsy.

Mechanism-resolving development provides a unifying design constraint: every consequential engineering claim should specify the biological state being controlled, the location of the control action, the credible alternative that addresses the same problem, the proximal tissue signal expected if the mechanism operates, the result that would falsify the claim and the action that follows when the limiting state changes. **Figure 1** contrasts outcome-only development with this mechanism-resolving chain.

**Figure 1:**
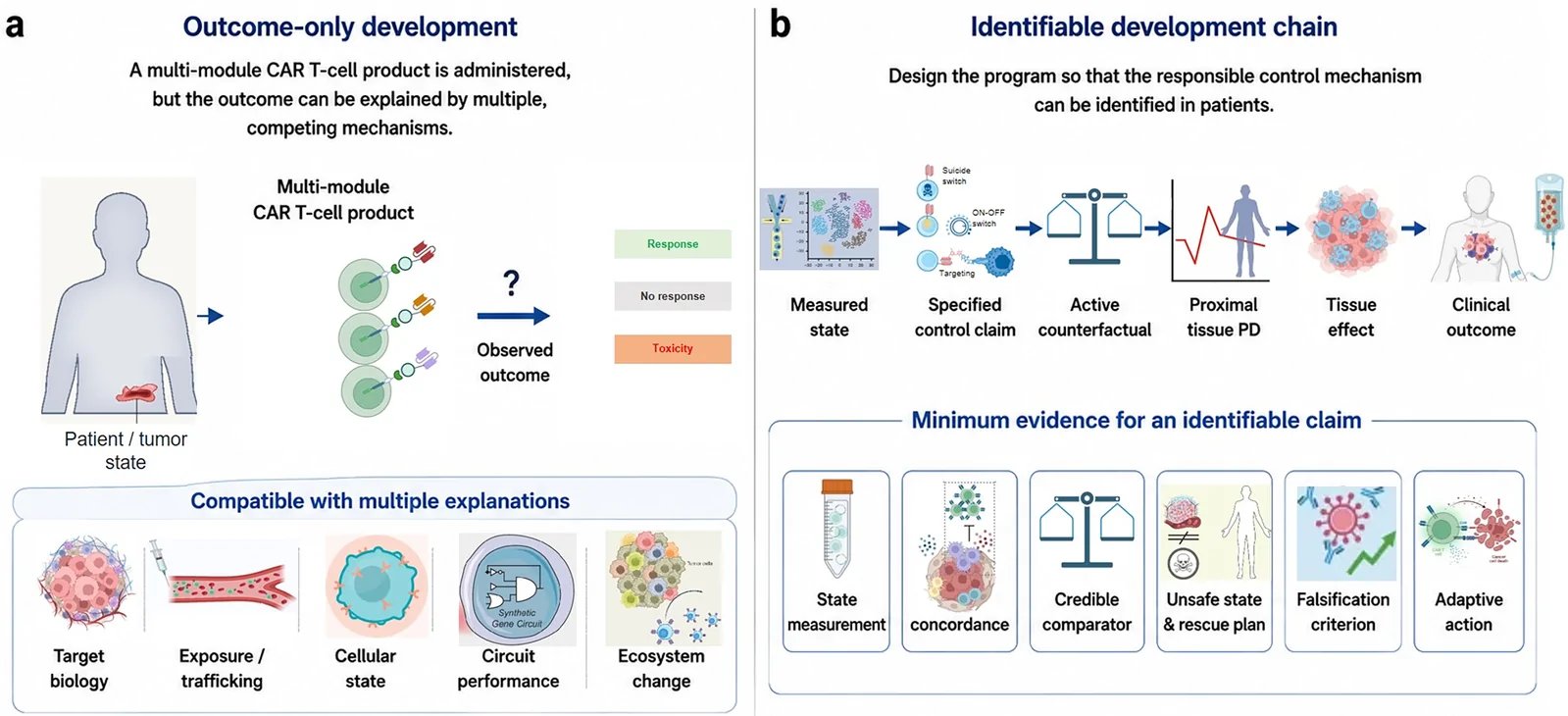
The mechanism-attribution gap in programmable solid-tumor CAR T-cell therapy. Panel **(a)** illustrates outcome-only development: a patient/tumor state is treated with a multi-module product, but response, non-response or toxicity remains compatible with target biology, exposure, cell state, circuit performance or ecosystem change. Panel **(b)** defines a mechanism-resolving chain from measured state and specified control claim through an active counterfactual, proximal tissue pharmacodynamics, tissue effect and clinical outcome. A defensible claim also requires temporal concordance, an unsafe-state and rescue plan, a falsification criterion and an adaptive action.

Mechanistic identifiability is used here in an operational clinical sense rather than as a synonym for formal structural or parameter identifiability in mathematical models (20, 21). It denotes the strength with which an observed outcome can be attributed to a prespecified control claim through relevant state measurement, temporal concordance, perturbation or comparison, proximal pharmacodynamics and exclusion of credible alternatives. This definition does not convert uncontrolled early-phase studies into causal experiments; it establishes a higher evidentiary standard than *post hoc* mechanistic narrative.

### 1.2 Four linked requirements for mechanism-resolving development

State resolution begins with the variable that the therapeutic system is intended to encounter or change. The relevant state may be insufficient antigen coverage, tumor-normal density overlap, inadequate tissue exposure, loss of functional competence, a suppressive cytokine pathway, a myeloid ecosystem state or a spatially restricted priming signal. The assay should approximate the variable sensed by the therapeutic cell and capture clinically important variation across lesions and time; a single archival biopsy is rarely sufficient.

Control-action resolution requires an explicit choice of where the therapeutic decision resides. Control can be placed in eligibility, route and schedule, receptor affinity or signaling, cell-state engineering, autonomous logic, an extrinsic ecosystem intervention or clinician-operated regulation. These locations are alternatives as well as complements. Shared-antigen risk, for example, can be addressed by diagnostic exclusion, affinity tuning, density filtering, genotype-conditioned veto, regional exposure, an adaptor or an elimination mechanism.

An active counterfactual is required to determine whether the selected control location is necessary. A logic circuit should not be compared only with an intentionally weak conventional CAR; it should be compared with a clinically credible lower-burden strategy aimed at the same limitation, such as quantitative eligibility, affinity tuning or external control. A fitness edit should be compared with manufacturing changes that preserve a favorable state without additional genomic alteration, and a hypoxia-regulated payload with regional or separately dosed local intervention.

Tissue-transition resolution requires a proximal pharmacodynamic readout in the compartment in which the claim operates. A route claim requires compartmental exposure and target engagement; a density claim requires quantitative tumor and normal-tissue distributions linked to output; a temporal circuit requires evidence of priming, induced product and decay; and ecosystem rewriting requires spatial evidence of perfusion, penetration, myeloid conversion, endogenous recruitment or antigen spreading. Peripheral expansion is informative but cannot substitute for tissue function.

Failure resolution requires prespecified falsification and adaptive action. The protocol should define the result that invalidates the proposed mechanism, the unsafe state that triggers intervention and the next decision if the original limitation is relieved but another becomes limiting. Treatment can unmask a pre-existing limitation, select a resistant state, induce inflammatory or functional failure, or relocate disease to an untreated compartment; these transitions should be measured separately rather than collapsed into a generic category of resistance.

Total-system burden is multidimensional. Vector size and gene edits create genetic burden; cell yield, stability and comparability create manufacturing burden; transition-state assays create analytical burden; and catheters, companion drugs, local sampling and rescue procedures create clinical-operational burden. These dimensions are not naturally commensurable. An architecture is dominated when another credible option is at least as favorable across all prespecified benefit, safety, observability, reversibility and feasibility dimensions and more favorable in at least one, taking uncertainty in the available evidence into account. **Figure 2** formalizes the control-allocation process, whereas **Table 1** operationalizes these requirements across representative control claims by linking the patient-relevant state, control location, active counterfactual, mechanism-proximal tissue pharmacodynamics, falsifying result and subsequent adaptive action.

**Figure 2:**
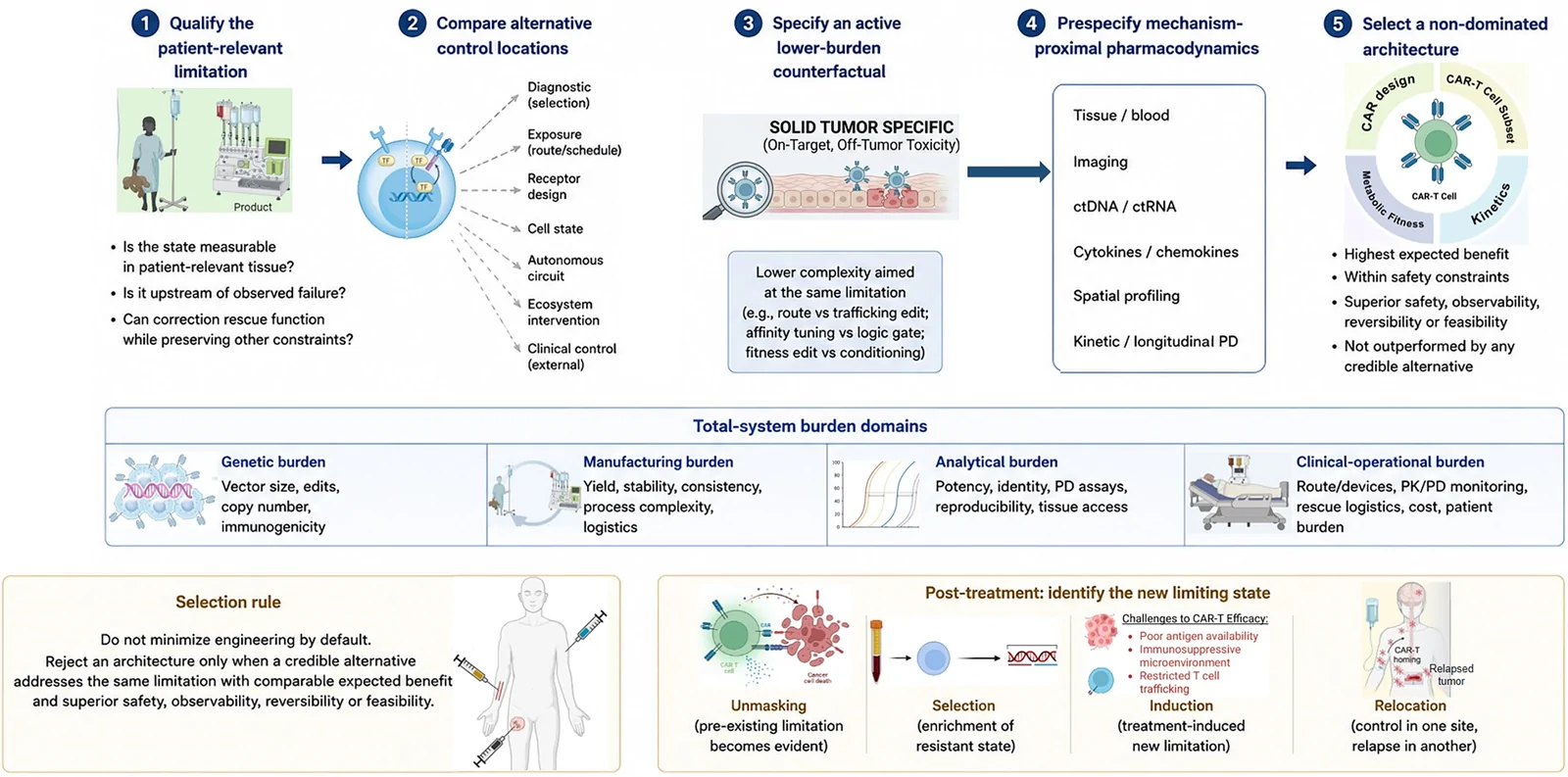
Building a mechanism-resolving product-trial system. A patient-relevant limitation is qualified before alternative control locations are compared. The selected architecture is tested against an active lower-burden comparator, supported by mechanism-proximal pharmacodynamics and evaluated across genetic, manufacturing, analytical and clinical-operational burden. An architecture is dominated when another credible option is at least as favorable across all prespecified benefit, safety, observability, reversibility and feasibility dimensions and more favorable in at least one, taking uncertainty in the available evidence into account. Post-treatment analysis distinguishes failure unmasking, selection, induction and anatomical relocation.

**Table 1:** Mechanism-resolving control claims: state definition, active counterfactuals, tissue pharmacodynamics and falsification.

Control claim	Patient-relevant state to resolve	Control location and active counterfactual	Mechanism-proximal tissue PD	Falsifying result and adaptive action	Refs
Antigen-density discrimination	Quantitative surface-density distributions in tumor and relevant normal tissues, including lesion-to-lesion and temporal variation	Density-sensitive receptor design versus quantitative eligibility, affinity tuning or route containment directed at the same tumor–normal discrimination problem	Local activation and tissue injury related quantitatively to measured antigen density and the predefined transition band	Tumor and normal-tissue distributions substantially overlap within the functional transition band → revise eligibility, target, receptor threshold or exposure strategy	(22, 23)
Temporal priming	Spatial distribution of the priming antigen together with induction, persistence and decay kinetics of the induced receptor or payload	IF–THEN circuit versus constitutive or rapid coincidence-based recognition addressing the same spatial-specificity requirement	Temporal linkage among priming input, induced product and local effector output, including serial re-priming and decay	Persistent leakage or function after departure from the authorized compartment → shorten functional memory, alter circuit kinetics or relocate control	(24–27)
HLA-loss activator–blocker control	Germline HLA genotype, tumor-specific LOH, activating-antigen density and intrapatient LOH heterogeneity	Genotype-conditioned activator–blocker logic versus diagnostic restriction, affinity tuning or regional exposure	Selective engagement of HLA-loss tumor while preserving HLA-retaining cells, with local assessment of activator and blocker performance	Heterogeneous or false LOH, inadequate inhibition or blocker saturation → exclude, retune or contain exposure	(28–32)
Hypoxia-regulated output	Oxygen-state distributions in malignant and clinically relevant nonmalignant tissues	Environment-responsive expression versus regional delivery or separately dosed local intervention	Oxygen-linked induction, localization of output and decay after reoxygenation	Normoxic leakage or activation in ischemic, inflamed or otherwise nonmalignant hypoxic tissue → externalize, pharmacologically control or discontinue the module	(17, 33)
Cell-state enhancement	Serial dysfunction, differentiation and functional competence under tumor-relevant stress	Genomic or transcriptional cell-state engineering versus manufacturing changes that preserve a favorable state without an additional permanent edit	Tissue persistence, phenotype and serial functional competence rather than circulating abundance alone	Expansion without tissue control, loss of productive function or excessive inflammatory activity → modify manufacturing, dose/schedule, supportive control or rescue	(34–43)
Ecosystem rewriting	Spatial myeloid, vascular, matrix or recruitment state that is proposed to be limiting	Cell-encoded ecosystem intervention versus a separately dosed tumor-microenvironmental therapy	Demonstration of the intended local state transition, such as myeloid conversion, improved perfusion or penetration, endogenous recruitment or antigen spreading	Payload expression without the predicted ecosystem transition, or unacceptable edema/vascular injury → externalize, contain or abandon the module	(44–51)
Regional exposure	Anatomical disease reservoirs, tissue accessibility and spatial distribution of disease	Locoregional or staged administration versus optimized systemic dosing and trafficking	Compartment-specific cell kinetics, target engagement, local pharmacodynamics and toxicity	High local exposure without control of relevant reservoirs or emergence of disease in an untreated compartment → adopt hybrid, staged or systemic exposure	(17, 18, 52–55)

### 1.3 Clinical maturity, mechanism attribution and evidence direction

Clinical evidence is often arranged on a single maturity ladder, although clinical advancement and mechanism attribution answer different questions. A randomized trial can establish comparative benefit while remaining weak evidence for the contribution of a particular module. Conversely, a severe toxicity can provide important evidence of normal-tissue injury, although the strength of the proposed recognition mechanism depends on the supporting anatomical and biological observations. Evidence should therefore be described along three orthogonal dimensions: clinical maturity, mechanism-attribution strength and direction.

Clinical maturity ranges from patient-relevant preclinical studies through first-in-human feasibility and quantitative early clinical evidence to comparative or regulatory evidence. Mechanism-attribution strength ranges from outcome-only observation to exposure linkage, proximal tissue pharmacodynamics, temporal perturbational concordance and component-level comparison. Evidence direction distinguishes observations that support a proposition, reveal a material trade-off or constrain the proposition. Attribution is assessed for a specified study-claim pair: evidence that cells reached a lesion, that a tissue state changed, and that an added module caused that change are distinct inferences. A comparison between differently engineered cohorts is informative but does not remove confounding automatically; randomization, matching and differences in manufacturing, conditioning and dose must be considered separately.

This separation prevents two recurrent errors: treating clinical advancement as proof that an engineered mechanism caused benefit, and relegating negative trials or toxicities to a lower evidentiary tier. EGFRvIII antigen reduction after tumor trafficking, fatal pulmonary toxicity after ERBB2 CAR-T administration and CEA-associated colitis are clinically adverse but mechanistically informative boundary conditions (56–58). **Figure 3** presents a qualitative rubric and explicit uncertainty rather than exact coordinates as objective measurements.

**Figure 3:**
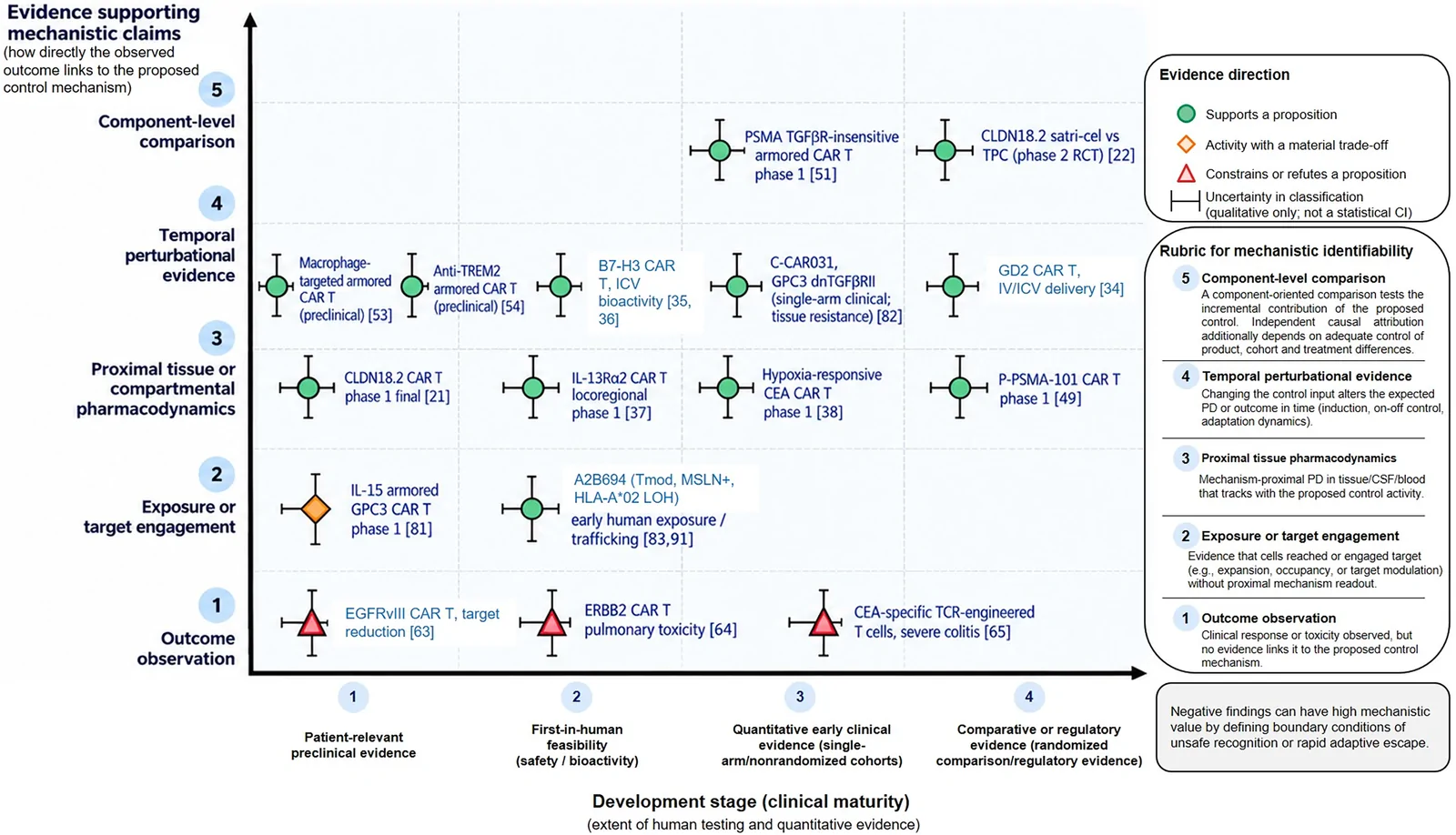
Development stage and evidence supporting mechanistic claims in engineered T-cell studies. The horizontal axis distinguishes patient-relevant preclinical evidence, first-in-human feasibility, quantitative early clinical evidence, and comparative or regulatory evidence. Quantitative early clinical evidence includes single-arm studies and nonrandomized clinical cohorts. The vertical axis distinguishes outcome observation, exposure or target engagement, proximal tissue or compartmental pharmacodynamics, temporal perturbational evidence, and component-level comparison. Each plotted point represents a specified study–claim pair, and the two axes are assigned independently: the horizontal position reflects clinical-development maturity, whereas the vertical position reflects the strength of evidence supporting the named mechanistic proposition. Trial phase, cohort size, randomization, regulatory status, or clinical activity therefore does not by itself confer stronger mechanistic attribution. Tissue detection establishes exposure but does not by itself establish receptor engagement, tissue-level control activity, or the incremental contribution of an added module. A randomized comparison of an integrated treatment package can establish comparative clinical benefit without isolating the contribution of an individual product or schedule component, whereas a nonrandomized component-oriented comparison can provide stronger component-level information but remains vulnerable to differences in cohort composition, manufacturing, dose, conditioning, and treatment context. The macrophage-directed studies (44, 45) are preclinical examples of local ecosystem changes and are not human clinical trials. C-CAR031 (59) contributes single-arm clinical and tissue-resistance evidence without an isolated clinical comparison of dnTGFβRII. A2B694 (32, 60) contributes early human exposure and trafficking evidence, not blocker-specific clinical attribution. The CEA-specific TCR-engineered study (58) is included as a shared-antigen safety boundary and is not a CAR-T trial. Green circles denote observations supporting the stated claim, orange diamonds denote activity accompanied by a material trade-off, and red triangles denote observations constraining or refuting the claim. Horizontal and vertical bars indicate qualitative uncertainty in classification, not statistical confidence intervals. The categories are author-defined and are not a validated evidence score. Clinical interpretation and denominators are specified in **Table 2**; preclinical observations are not counted as human clinical cohorts.

**Table 2:** Study-level clinical outcomes, safety findings and limits of mechanistic attribution.

Study/setting	Population and activity	Major safety findings	Evidence supported	Unresolved inference	Refs
CT041/satri-cel, phase 1, gastrointestinal cancers	98 treated. ORR 38.8%; DCR 91.8%. Median PFS 4.4 months; OS 8.8 months. The cohort included monotherapy and combination/sequential-treatment groups.	CRS in 95/98, all grade 1–2; gastric mucosal injury in 8/98. No reported DLT, treatment-related death or ICANS.	Activity and cellular kinetics of a biomarker-selected treatment package.	Contributions of antigen threshold, conditioning, manufacturing, combination treatment and repeat infusion are not independently isolated.	(61)
Satri-cel versus physician-choice therapy, randomized phase 2	156 randomized: 104 versus 52; 88 versus 48 received treatment. ITT median PFS 3.25 versus 1.77 months; HR 0.37, 95% CI 0.24–0.56.	Grade ≥3 TEAEs in 87/88 versus 30/48. CRS in 84/88 satri-cel recipients. Safety and ITT populations differ.	Comparative benefit of the complete treatment strategy.	Randomization does not identify the independent contribution of a product module, conditioning or reinfusion schedule.	(62)
GD2 CAR-T, H3K27M-mutant diffuse midline glioma	13 enrolled; 11 received IV cells; 9 subsequently received ICV cells. Major volumetric reductions in 4/11, with smaller reductions in 3 others. These are not a pooled RECIST ORR.	IV CRS in 11/11; grade 4 CRS/DLT in 3/11. ICANS in 5/11, including one grade 3 event. Grade ≥3 TIAN in 5/9 IV-treated DIPG patients. No DLT during 62 ICV infusions.	Compartmental pharmacology and longitudinal biological activity.	Route, dose, repeated treatment and selection for subsequent ICV administration remain confounded. Neuroinflammatory toxicity is relevant to interpretation of the treatment strategy.	(52)
B7-H3 CAR-T, DIPG, BrainChild-03	23 enrolled; 21 treated; 18 imaging-evaluable. PR 1/18; SD 15/18; progressive disease 2/18. Median OS from first infusion 10.7 months.	One grade 4 intratumoral hemorrhage was the sole DLT: 1/18 DLT-evaluable and 1/21 treated. Grade 3 hydrocephalus in 1/21; no observed ICANS.	Serial ICV administration and CSF pharmacodynamic observations.	No randomized route comparison; preliminary and expanded publications overlap. Spatial coverage, local toxicity and radiographic interpretation remain relevant limitations.	(53, 54)
IL-13Rα2 CAR-T, recurrent high-grade glioma	65 treated; 58 response-evaluable after ≥3 infusions. SD or better in 29/58; PR in 2/58 and on-protocol CR in 1/58. An additional off-protocol CR is not added to that numerator.	Grade ≥3 events with possible or greater CAR-T attribution in 23/65. Two grade 4 cerebral-edema events had possible attribution. One grade 3 encephalopathy and one grade 3 ataxia had probable attribution. No DLT.	Serial CSF/tumor-cavity-fluid cytokine and cellular measurements.	Local inflammation is not specific proof of tumor killing. Route, manufacturing and schedule evolved across study arms.	(18)
PC13, hypoxia-responsive CEA CAR-T	43 treated: IP 17, IV 26. Response-evaluable: IP 17, IV 25. ORR 4/17 versus 2/25; DCR 14/17 versus 17/25.	Grade 3 diarrhea in 9/43; grade 1–2 CRS in 33/43.	Exposure and activity with a hypoxia-responsive construct administered through nonrandomly assigned routes.	No constitutive-CAR comparator isolates hypoxia regulation; route comparisons are confounded by disease distribution and cohort differences.	(17)
Conventional versus IL-15-armored GPC3 CAR-T	12 conventional-CAR and 12 15.CAR recipients. ORR 0/12 versus 4/12; 15.CAR DCR 8/12. Selected dose-matched comparisons used 6 conventional-CAR recipients.	CRS requiring immunomodulatory treatment in 9/12 15.CAR versus 1/6 dose-matched conventional-CAR recipients. iCasp9 activated in 3/12 15.CAR recipients: one prolonged grade 3 CRS, one prolonged grade 2 CRS, and one respiratory deterioration requiring mechanical ventilation.	Nonrandomized component-oriented comparison with concordant expansion and tumor-biopsy observations.	Product, cohort and dose differences limit independent attribution to IL-15. Safety-switch activation is not equivalent to a cohort-wide grade ≥3 CRS rate.	(43)
C-CAR031, GPC3/dnTGFβRII, hepatocellular carcinoma	36 treated. ORR 16/36; tumor regression in 32/36. Median PFS 4.2 months; OS 14.2 months.	CRS in 34/36, including grade 3 CRS in 2/36. Nonhematological grade ≥3 adverse events in 9/36.	Single-arm activity and analyses supporting GPC3 loss and increased TGFβ as candidate resistance states.	No clinical component comparator isolates dnTGFβRII. Resistance-state evidence does not establish that the module caused initial benefit.	(59)
A2B694 Tmod, MSLN-positive/HLA-A*02-LOH tumors	Phase 1 cohort: 13 treated; best responses CR 1/13, SD 8/13 and progressive disease 4/13. ASCO 2026 poster: tumor detection in 3/3 available DL2 biopsies and 1/1 DL3 biopsy.	Grade 3 ICANS in 1/13; grade 1 CRS in 1/13, with IL-2 confounding reported. No reported DLT.	Preliminary human exposure and trafficking observations.	Small heterogeneous conference-reported cohort; independent blocker contribution and protection of HLA-retaining tissue are unestablished. CNS relapse during non-CNS CR is an anatomical observation, not proof of its cause.	(32, 60)
PSMA CAR-T with dominant-negative TGFβ receptor, mCRPC	18 enrolled; 13 treated. One patient had >98% PSA reduction; three additional patients had ≥30% reductions, giving 4/13 in total. PSA changes are not RECIST responses.	Grade ≥2 CRS in 5/13. One patient developed grade 4 CRS with concurrent sepsis and died.	Blood expansion, tumor trafficking and paired-tissue analyses.	No matched clinical PSMA-CAR comparator lacking the resistance module; neither benefit nor the severe inflammatory/infectious event isolates its contribution.	(19)
P-PSMA-101, mCRPC	33 treated. PSA50 in 7/33; RECIST PR in 1/13 evaluable patients.	DLTs in 6/33; CRS in 20/33, including grade ≥3 in 3/33. Safety switch used in 8/33; seven toxicities resolved and one event was fatal.	Clinical expansion/activity and safety-switch intervention.	The contribution of the manufactured TSCM-rich state is not isolated. Rescue intervention is a different mechanistic claim from cell-state engineering.	(42)
EGFRvIII CAR-T, recurrent glioblastoma	10 treated with a single IV dose. Post-treatment surgery in 7/10; EGFRvIII reduction in 5/7 assessed tumors. One patient had residual SD for >18 months.	No reported CRS or off-tumor toxicity in this small cohort.	Tumor access, target reduction and adaptive local changes.	Selective tissue sampling limits inference; tissue access and antigen reduction do not establish durable benefit or isolate every resistance mechanism.	(56)
ERBB2 CAR-T, single-patient safety report	Respiratory distress and pulmonary infiltrates occurred within 15 minutes; death occurred 5 days later. No meaningful efficacy estimate.	Fatal inflammatory/pulmonary event in the reported patient.	A treatment-associated safety observation requiring explanation.	Normal-lung ERBB2 recognition was the investigators’ proposed explanation, not an independently isolated pathway. A case report does not estimate population incidence.	(57)
CEA-specific TCR-engineered T cells, metastatic colorectal cancer	3 treated. Objective tumor regression in 1/3; serum CEA declined 74–99% in 3/3.	Severe transient inflammatory colitis in 3/3, constituting a DLT.	Shared-antigen safety evidence from a TCR-engineered platform.	The study informs shared-antigen targeting risk but does not test a CAR architecture or a CAR-specific control module.	(58)

Treated, response-evaluable, randomized, DLT-evaluable and infusion-level populations are distinct. Response measures are study-specific; volumetric changes, RECIST responses, PSA declines and disease control are not interchangeable. Safety denominators are treated patients unless otherwise indicated. Treatment-emergent events, treatment-attributed events and events requiring intervention are not equivalent classifications. Safety-switch use does not establish adverse-event grade. References 35 and 36 report overlapping BrainChild-03 populations and are not counted as independent cohorts. A2B694 remains conference-reported evidence; biopsy denominators follow the poster (60). Preclinical studies (44, 45) are discussed separately and are not included as human clinical cohorts. CR, complete response; CRS, cytokine release syndrome; DCR, disease control rate; DIPG, diffuse intrinsic pontine glioma; DL, dose level; DLT, dose-limiting toxicity; ICANS, immune effector cell-associated neurotoxicity syndrome; ICV, intracerebroventricular; IP, intraperitoneal; ITT, intention-to-treat; IV, intravenous; mCRPC, metastatic castration-resistant prostate cancer; ORR, objective response rate; OS, overall survival; PFS, progression-free survival; PR, partial response; PSA50, PSA decline of at least 50%; SD, stable disease; TEAE, treatment-emergent adverse event; TIAN, tumor inflammation-associated neurotoxicity.

### 1.4 Recognition architectures are distinct causal claims

Programmable recognition systems are frequently grouped under the label logic-gated CAR, but they implement different computations. OR-gated and tandem receptors broaden the recognition surface by allowing either target to activate the cell. They can reduce antigen-negative escape but expose the product to the union of both normal-tissue expression patterns. Their critical assays therefore include each single-positive state, the double-positive state, unequal density, mixed populations and longitudinal target evolution.

AND systems require coincident positive inputs. Cooperative proximal-signaling systems such as LINK CARs implement rapid dual-input dependence, whereas transcriptional systems introduce a time-dependent state (63). The clinically relevant variable is not simply co-expression somewhere in the tumor; it is same-cell or spatial co-expression at densities compatible with the mechanism, together with maximum permissible single-positive leakage.

SynNotch-like IF-THEN systems use a priming encounter to induce a second receptor or payload (24–27). Their behavior cannot be represented adequately by a static truth table because the output state depends not only on the current antigen combination but also on prior priming, persistence of the induced program and its subsequent decay. In secondary-CAR circuits, this history dependence permits a finite interval during which the priming antigen is no longer encountered but the induced CAR remains available for downstream-antigen recognition; the duration and functional consequences of that interval require circuit-specific measurement. Induction time, output amplitude, persistence after priming, serial re-priming, migration during the functional window and decay determine both efficacy and off-site risk. A spatially restricted priming antigen can support trans-killing of neighboring heterogeneous tumor cells, but carryover beyond the intended territory is a specific unsafe state.

Antigen-density filtering is a continuous transfer-function problem rather than a binary gate (22, 23). A steep response curve in engineered cell lines is insufficient. Clinical usefulness requires a reproducible gap between tumor and relevant normal-tissue density distributions, a defined transition band, concordance between the eligibility assay and surface density, and robustness to receptor-expression drift, inflammation and product state.

Inhibitory CARs and HLA-loss activator–blocker systems integrate activating and inhibitory inputs rather than implementing an unconditional Boolean veto (28–31, 64). Their output depends on receptor design, antigen abundance and cellular context; incomplete inhibition under relevant input combinations is a potential failure mode to test rather than a universal property of all architectures. HLA-loss Tmod additionally requires genotype-defined selection: the patient must carry the protective HLA allele, the tumor must show the relevant allele-specific loss and the activating antigen must remain available. Loss of the protective ligand removes an inhibitory input; it does not itself establish adequate tumor exposure, productive activation or normal-tissue protection. Early A2B694 data describe systemic persistence, tumor detection at higher dose levels and one confirmed systemic complete response in a small conference-reported cohort; these observations do not independently demonstrate blocker function in patients (32). Logic architectures can also be layered rather than deployed as isolated control modes. For example, in preclinical glioblastoma models, an EGFRvIII-sensing synNotch receptor was used as an IF-THEN priming layer to induce an EphA2/IL13Rα2 tandem CAR, thereby coupling spatially restricted authorization to OR-like downstream recognition of heterogeneous neighboring tumor cells (25). More complex transcriptionally linked receptor systems have similarly combined positive and negative logic or serial and parallel multi-input recognition to implement AND-NOT and OR-AND decision rules (65). Such composite architectures should be treated as new causal claims rather than as the simple sum of their constituent gates. Priming kinetics, antigen density, receptor induction and decay, veto strength, migration and input sequence can interact to generate intermediate or unsafe states that are not predicted from testing each module independently. Validation should therefore interrogate the joint input-state space, including the order and duration of inputs, intermediate states, off-condition leakage and failure of one control layer while the remaining layers remain functional. A cytotoxicity endpoint alone cannot establish that an individual control module or a composite circuit operated as intended; validation must also resolve the relevant inputs, intermediate states and off-condition behavior. **Figure 4** illustrates their distinct computational representations, whereas **Table 3** links each architecture to the transition-state tests, candidate potency measures, extended characterization, tissue biomarkers, unsafe states and control strategies required to determine whether the intended mechanism actually operated.

**Figure 4:**
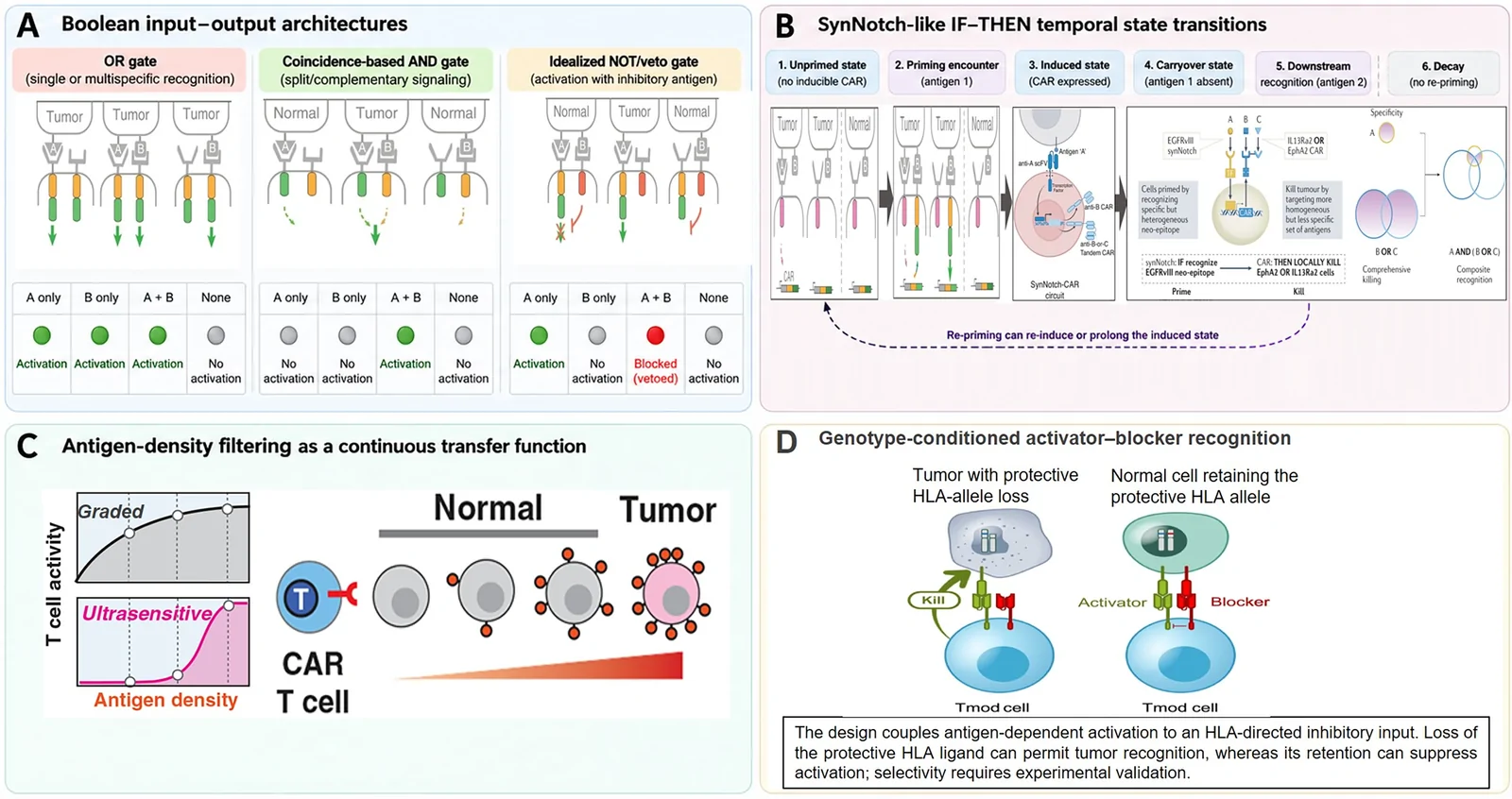
Recognition architectures require mechanism-specific representations and assays. **(a)** Boolean OR, coincidence-based AND, and idealized NOT/veto architectures are represented by discrete input–output states. OR recognition permits activation through either antigen input, whereas the coincidence-based AND example requires complementary positive inputs from antigens A and B for productive activation. The idealized single-positive AND states are non-permissive, although experimentally measurable single-input leakage must be assessed. The NOT/veto example illustrates the qualitative consequence of a protective or inhibitory input that suppresses otherwise activating recognition. **(b)** SynNotch-like IF–THEN systems couple priming-antigen recognition to induction of a secondary receptor or effector program. The numbered headings identify conceptual stages from the unprimed state through induction, carryover, downstream recognition and decay; the receptor diagrams illustrate example implementations rather than measured kinetic snapshots. In the secondary-CAR implementation, the induced receptor can persist transiently after the priming antigen is no longer encountered, allowing downstream-antigen recognition during a finite carryover interval (24–27). Effector activation during this interval remains dependent on the downstream antigen and is not antigen-independent killing. Renewed priming can re-induce or prolong the induced state. The EGFRvIII-synNotch–EphA2/IL13Rα2 example combines temporal priming with broader downstream recognition; its composite recognition notation is not a static requirement for simultaneous expression of all antigens on the same target cell (25). **(c)** Antigen-density filtering is illustrated by graded and ultrasensitive input–output relationships (22, 23). The target-cell cartoons depict a favorable example of lower antigen density in normal tissue and higher density in tumor tissue; they do not establish a universal tumor–normal separation. The curves are illustrative and do not represent measured clinical thresholds or population distributions. **(d)** The Tmod example illustrates a genotype-conditioned implementation of activator–blocker recognition. An antigen-directed activating receptor is paired with an inhibitory receptor recognizing a protective HLA ligand. In an appropriately selected HLA-heterozygous patient, tumor-specific loss of the protective allele can remove the inhibitory ligand while retaining the activating antigen, whereas normal cells retaining the protective HLA ligand can engage the blocker (28–31). The illustration represents the intended recognition principle, not established clinical protection of all HLA-retaining tissues. Unlike the abstract input–output states in panel a, panel d identifies the biological basis for genotype-dependent discrimination. Activator and blocker function must be tested jointly across relevant antigen densities and cellular contexts.

**Table 3:** Mechanism-specific decision architectures, transition-state assays, product characterization and clinical control.

Architecture or control mode	Correct mechanistic representation and claim	Transition-state/candidate potency tests	Extended characterization and tissue biomarker	Unsafe state or falsifying clinical PD	Control or rescue	Refs
Baseline target recognition and proximal potency	Target-dependent recognition and proximal effector activity	Identity, viability, CAR expression and target-dependent activation using defined reference cells	Quantitative density series, serial challenge, donor/lot variability, receptor-expression variation and relevant normal-tissue panels	Tissue exposure without productive engagement; activation against low-density essential normal tissue	Dose interruption, standard organ-specific toxicity management and elimination when justified	(66–68)
OR/tandem recognition	Boolean coverage in which either antigen can permit activation; intended to broaden recognition and reduce antigen-negative escape	Each single-positive state, the double-positive state, unequal antigen densities and mixed populations	Longitudinal target mapping across lesions together with assessment of both relevant normal-tissue expression patterns	Activity against either normal-tissue antigen distribution or progressive escape outside the broadened recognition space	Eligibility or exposure restriction; receptor redesign when expanded coverage creates unacceptable normal-tissue recognition	(3, 8, 12)
AND/LINK coincidence	Coincident positive inputs or cooperative proximal signalling; intended to derive specificity from co-expression	All permissive and non-permissive combinations, same-cell versus spatial co-expression, unequal densities and repeated decisions	Input state linked quantitatively to local CAR output under clinically plausible antigen distributions	Single-positive leakage, failure at density imbalance or loss of dependence on coincident inputs	Eligibility restriction, route containment, circuit retuning or rapid suppression/elimination where available	(63)
NOT/inhibitory CAR	Activator–veto decision rather than simple Boolean absence; protective input should suppress activation	Activating-input series crossed with inhibitory-marker density, including partial marker loss, inflammation and repeated challenge	Local activating and protective-marker abundance linked to output and tissue injury	Injury despite the protective marker, incomplete inhibition or veto saturation at high activating input	Exposure containment, pharmacological suppression where available or elimination	(64)
IF–THEN/synNotch-like temporal control	Temporal state-transition system in which a priming event induces a second receptor or effector program	Induction time, amplitude, serial priming, migration, carryover, decay and off-state leakage	Spatial measurement of priming antigen, induced product and local output during and after the functional window	Prolonged off-site activity, persistent induced product after leaving the priming territory or insufficient decay	Shorten functional memory, change priming architecture or relocate control to a more reversible layer	(24–27)
Antigen-density filter	Continuous transfer function with a predefined transition band rather than a binary threshold	Response curve across calibrated surface densities; lot/receptor-expression variation	Concordance between eligibility assay and quantitative surface density; inflammatory induction in relevant normal cells	Tumor–normal overlap within the functional transition band or clinically meaningful threshold drift	Revise eligibility, contain route/exposure or retune receptor sensitivity	(22, 23)
HLA-loss activator–blocker	Genotype-conditioned integration of activating and inhibitory inputs, intended to distinguish HLA-loss tumor cells from HLA-retaining normal cells; joint input–output behavior and selectivity require experimental validation	Activator potency and blocker function in HLA-retaining and HLA-loss reference cells across variable antigen/HLA density	Patient-matched mixed-cell systems, LOH heterogeneity, incomplete LOH and blocker saturation	False or heterogeneous LOH, loss of blocker function or injury to HLA-retaining tissue	Exclude ineligible patients, modify exposure or activate elimination control	(28–32)
Hypoxia-regulated output	Environmental sensing in which low oxygen authorizes CAR or payload expression	Minimum hypoxic induction, maximal normoxic leakage and response across oxygen gradients	Reoxygenation kinetics, nonmalignant hypoxia and spatial payload localization	Activation in ischemic or inflamed normal tissue, persistent normoxic expression or inadequate decay	Regional containment, pharmacological gate where available, elimination and organ-specific rescue	(17, 33)
Functional-competence engineering	Preservation or redirection of productive T-cell state under serial tumor challenge	Robust proximal function linked to serial activation, killing or polyfunctionality	Repeated stimulation, metabolic stress, differentiation/exhaustion state, clonality and long-term safety	Persistent circulating expansion without tissue control, dysfunctional persistence or clonal dominance	Fractionated dosing, pharmacological suppression/rest or elimination where appropriate	(35, 37, 40, 42, 43, 59)
Ecosystem rewriting	Engineered output intended to alter a defined myeloid, vascular, matrix or recruitment state	Module-appropriate target-engagement or functional output assay	Spatial penetration, perfusion, myeloid conversion, endogenous recruitment and antigen spreading	Detectable payload without the proposed tissue-state transition; edema, vascular leak or pathological remodeling	Regional containment, inducible output, separately dosed intervention or organ-directed rescue	(44–51)
External adaptor or drug control	Clinician-operated, pharmacologically regulatable activity	Target-dependent function across drug/adaptor concentration, including a defined off-state ceiling	PK–PD, tissue penetration, on/off and washout kinetics, immunogenicity and repeated cycling	Residual activity despite withdrawal, inadequate tissue penetration or inability to regain control after cycling	Withdraw or antagonize the controlling drug/adaptor; eliminate the cells if control is irreversible or inadequate	(69–74)

### 1.5 Exposure and functional competence are co-prerequisites

Route is a pharmacological variable because it determines where the cell encounters antigen, where inflammation is concentrated, whether repeated dosing is feasible and which biological samples can be obtained. Regional administration can bypass vascular or blood-brain-barrier limitations, but it can also create catheter complications, edema, hemorrhage or concentrated tissue injury. A route claim therefore requires evidence that relevant disease reservoirs were exposed and that risk was not merely shifted to another compartment.

Central nervous system programs provide the clearest examples. Staged intravenous and intracranial GD2 CAR T-cell therapy in H3K27M-mutant diffuse midline glioma produced marked regressions in a subset while requiring intensive management of tumor inflammation-associated neurotoxicity (52). Repeated intraventricular B7-H3 dosing integrated serial exposure with cerebrospinal-fluid monitoring (53, 54). Locoregional IL-13Rα2 therapy linked repeated administration to cerebrospinal-fluid IFNγ, CXCL9 and CXCL10 (18). These studies support compartmental pharmacology and local observability; they do not isolate route as the sole cause of efficacy.

Peritoneal and pleural disease offer related opportunities. The hypoxia-responsive CEA study reported numerically higher response with intraperitoneal than intravenous administration, but route was not randomized and the cohorts were small (17). Regional mesothelin therapy demonstrated that local administration can coexist with systemic checkpoint blockade and durable immune effects in selected pleural disease (55). In both settings, a route-optimized conventional product is a necessary comparator before trafficking or circuit complexity is declared essential.

Exposure has value only if the cells remain functionally competent after arrival. Vector copies in blood and persistent CAR-positive cells do not establish serial killing, metabolic resilience or productive differentiation in tumor tissue. c-Jun, FOXO1, transient rest, BACH2 dosage and CARD11-PIK3R3 engineering illustrate distinct ways to preserve or redirect cell state (34–41). Their translational claim is not persistence in isolation, but productive and controllable function under repeated stimulation and tumor-like stress.

Recent clinical programs strengthen this distinction. In the IL-15 GPC3 program, conventional and IL-15-armored products were evaluated in separate nonrandomized cohorts of 12 patients each. Objective responses occurred in 0/12 and 4/12 patients, respectively, with greater expansion and tissue persistence in the IL-15 cohort; inflammatory toxicity sometimes required activation of inducible caspase-9 (43). This is a component-oriented clinical comparison, but cohort and product differences limit independent attribution to IL-15. C-CAR031 combined GPC3 recognition with dominant-negative TGFβRII and produced an objective response in 16/36 patients. Tumor analyses and functional studies implicated GPC3 loss and increased TGFβ in resistance, but the single-arm clinical study did not isolate the contribution of dnTGFβRII (59).

### 1.6 Context control is not one mechanism: sensing, resistance and ecosystem rewriting

The tumor microenvironment can be addressed in three mechanistically distinct ways. A sensing strategy uses a local cue to authorize an output. A resistance strategy prevents a suppressive cue from disabling the therapeutic cell. A rewriting strategy directly changes the ecosystem through the engineered cell or a separately administered intervention. Combining these categories under armoring obscures both the intended state change and the associated risk.

Hypoxia-responsive products illustrate sensing. Oxygen-regulated promoters or degradation domains can restrict CAR or payload expression to low-oxygen conditions (33). The CEA program establishes clinical manufacturability and feasibility but does not establish hypoxia as a tumor-exclusive signal (17). Ischemia, wound repair, inflamed mucosa and poorly perfused normal tissue are relevant counterexamples. Identifiability therefore requires measured oxygen state, induced output, normoxic leakage, reoxygenation decay and testing in nonmalignant hypoxic models.

Dominant-negative TGFβ receptors illustrate resistance. The receptor does not authenticate location; it preserves competence despite suppression. PSMA-directed TGFβ-insensitive CAR T cells expanded and produced activity in some patients; severe CRS and concurrent sepsis in one patient identify an important clinical safety concern, but do not isolate the contribution of TGFβ resistance to toxicity (19). C-CAR031 provides a more recent signal of activity in hepatocellular carcinoma, but its contribution must still be interpreted alongside target selection, product state, dose and conditioning (59).

Rewriting is favored when the ecosystem itself is the actionable state. IL-12-armored CAR T cells directed against FOLR2- or TREM2-expressing macrophages produced durable myeloid reorganization and endogenous immunity after contraction of the transferred cells in preclinical metastatic models (44, 45). Inflammatory monocytes can support intratumoral T-cell restimulation through chemokines, IL-15 and acquired peptide-MHC complexes (46). These observations make myeloid organization a candidate therapeutic target, not merely another contextual input.

Matrix, vascular and recruitment programs require equally specific endpoints. Heparanase should be evaluated by spatial penetration; IL-7/CCL19 by controlled recruitment and endogenous repertoire; PAK4 or localized VEGF blockade by perfusion and vascular state; and IL-10 expression by cell metabolism, dysfunction resistance and systemic inflammatory consequences (47–51). Serum cytokine elevation and rapid monolayer killing do not establish ecosystem conversion. The three forms of context control also create distinct safety failure modes and should not be interpreted as an intrinsic safety hierarchy. Sensing strategies can fail through false authorization when the contextual cue is shared by nonmalignant tissue, or through excessive persistence of the induced output after the permissive state has resolved; cue specificity, off-state leakage and decay kinetics are therefore central safety variables (17, 33). Resistance strategies do not authenticate tissue location but instead reduce suppressive restraint on activated cells, potentially amplifying antigen-driven activity and narrowing the inflammatory control margin; activation magnitude, reversibility and systemic inflammatory effects are therefore particularly important (19, 59). Ecosystem-rewriting strategies can generate non-cell-autonomous effects on myeloid, vascular, matrix or recruitment states, creating potential risks of excessive inflammation, edema, vascular leak or pathological remodeling beyond the immediate CAR T-cell-target interaction (44–51). Their safety assessment should therefore emphasize spatial containment, reversibility and evidence that the intended local tissue-state transition does not propagate into harmful organ-level remodeling. These are mechanism-based risks to test, not estimates of comparative clinical incidence; several ecosystem-rewriting examples remain preclinical (44–51).

### 1.7 Clinical evidence: benefit, attribution and boundary conditions

Human studies should be interpreted according to the proposition they actually test. The CLDN18.2 program provides randomized evidence that a biomarker-defined product-clinical package can improve progression-free survival. In the final phase 1 analysis, 98 patients were treated; objective response rate was 38.8%, disease control rate 91.8%, median progression-free survival 4.4 months and median overall survival 8.8 months; gastric mucosal injury occurred in 8.2% (61). In the randomized phase 2 study, satri-cel improved median progression-free survival to 3.25 months versus 1.77 months with physician-choice therapy (hazard ratio 0.37) (62). Regulatory approval in China in June 2026 established the first approved CAR T-cell product for a solid-tumor indication (75). These observations validate the integrated target-diagnostic-product package, not the contribution of repeated dosing or any untested additional circuit.

Regional central nervous system studies test a different proposition. GD2, B7-H3 and IL-13Rα2 programs provide distinct quantitative examples of staged or repeated CNS exposure, with study-specific differences in response, compartmental pharmacodynamics and neuroinflammatory toxicity (18, 52–54). Their mechanistic value increases when cell kinetics, cytokines, imaging and target evolution are aligned, but none provides a randomized route comparison.

Context, resistance and fitness programs provide early evidence with different levels of attribution. The same hypoxia-responsive CEA construct was administered through nonrandomized routes selected by disease distribution; a constitutively expressed comparator was not included, so neither route effects nor the incremental contribution of hypoxia regulation can be isolated (17). PSMA-targeting dnTGFβR CAR T cells and P-PSMA-101 are separate early clinical programs with antitumor activity and important inflammatory toxicity or safety-switch use (19, 42). The IL-15 GPC3 program includes a nonrandomized conventional-versus-armored cohort comparison, whereas C-CAR031 provides single-arm activity and tissue-based resistance analyses (43, 59). Early A2B694 data support systemic persistence and tumor trafficking in genotype-selected patients, but not independent clinical attribution to the blocker. The reported CNS relapse during ongoing non-CNS complete response illustrates a possible anatomical escape pattern, rather than proof of its cause (32).

Negative observations remain indispensable. EGFRvIII-directed cells reached glioblastoma tissue but were followed by target reduction and adaptive suppression (56). Rapid pulmonary toxicity and a subsequent death followed ERBB2 CAR-T infusion; recognition of low-level ERBB2 on normal lung epithelium was the investigators’ proposed explanation, not a directly isolated mechanism (57). CEA-specific TCR-engineered T cells produced tumor activity but induced severe transient colitis in all three treated patients (58). These observations demonstrate that clinical activity, tissue access and mechanistic attribution are related but non-equivalent forms of evidence. **Table 2** therefore separates the quantitative clinical signal from the inference supported by the study, the limits of mechanism attribution and the inference that remains unresolved.

### 1.8 Mechanism-adaptive trial development

Early cell-therapy trials should be mechanism-adaptive rather than dose-escalation-only. The central uncertainty should determine what each cohort measures and what the next cohort changes. When a threshold is uncertain, paired quantitative target measurements and output markers are required. When ecosystem rewriting is claimed, serial spatial profiling or validated surrogates should demonstrate the predicted state transition.

Mechanism-adaptive trials require coordinated development of the cellular product, enrollment assays and mechanism-proximal pharmacodynamic assays. A companion diagnostic provides information essential to the safe and effective use of a corresponding therapy; an exploratory pharmacodynamic assay does not automatically meet that definition (76). IHC and spatial proteomic assays should have an explicit scoring or image-analysis procedure and, for density or co-expression claims, a justified relationship to the surface state sensed by the CAR. HLA-loss strategies require germline HLA typing and tumor-specific LOH assessment with attention to tumor purity and intralesional heterogeneity. Before an assay drives enrollment or cohort adaptation, its performance should be established for that intended use, including specimen handling, analytical sensitivity, specificity, precision, inter-reader or inter-laboratory reproducibility and the relevant decision threshold (66, 76). The protocol should prespecify assay versions, sampling windows, cutoffs and handling of indeterminate results. Changes to a cutoff, platform or analysis pipeline between cohorts require documented bridging or reanalysis of retained specimens where feasible; otherwise an apparent biological change may instead reflect measurement drift. Exploratory assays may undergo staged, fit-for-purpose qualification, but results that remain analytically uncertain should not determine a mechanistic failure assignment or an adaptive treatment decision.

Active counterfactuals can be introduced without converting every phase 1 study into a large randomized trial. Options include route cohorts with matched eligibility, staggered introduction of a module after calibration of a conventional product, randomized allocation among biologically plausible dose levels, within-patient on-off control for adaptor systems or prospective component ablation in a preclinical-to-clinical bridge. Bayesian designs that model activity and toxicity jointly are better aligned with self-amplifying cell products than maximum-tolerated-dose assumptions alone (77, 78).

Mechanism attribution also requires lesion-level analysis. Different metastases can have different targets, exposure and ecosystem states, so the relevant unit may be a bottleneck set rather than a single patient-level limitation. A therapy can control systemic disease while a central nervous system reservoir escapes, or relieve exposure while selecting antigen-low clones. Longitudinal analysis should distinguish unmasking of a pre-existing limitation, selection of a resistant state, induction of a new toxicity or dysfunction and anatomical relocation to an untreated compartment.

**Figure 5** applies the same decision process to three settings. CLDN18.2-positive gastric cancer initially prioritizes quantitative eligibility, mucosal safety and compartmental exposure. Central nervous system programs prioritize route, local pharmacodynamics, neuroinflammation and repeated functional competence. MSLN-positive tumors with HLA-A*02 loss create a setting in which genotype-conditioned activator-blocker logic can be more rational than affinity, route or diagnostic restriction alone. This third setting shows that the design principle is not anti-complexity; autonomous logic is justified when the required malignant state cannot be reproduced by simpler control locations.

**Figure 5:**
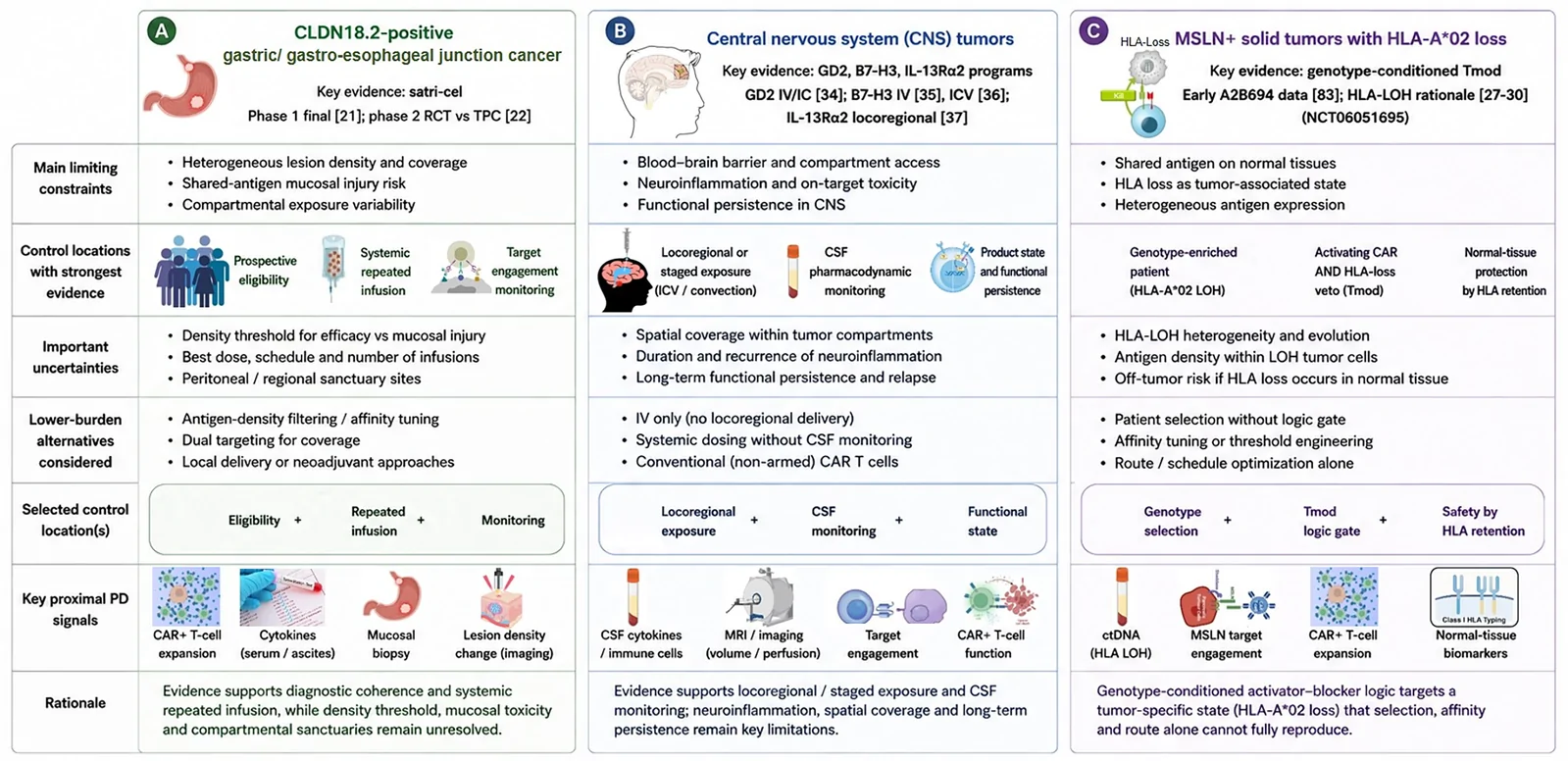
A common framework compares control locations across disease contexts. In CLDN18.2-positive gastric or gastro-oesophageal junction cancer, clinical studies support biomarker-selected treatment strategies, while the independent contributions of selection thresholds, repeated dosing and compartmental exposure remain incompletely resolved (61, 62). CNS programs provide examples of locoregional or sequential IV–ICV administration with compartmental monitoring, alongside neuroinflammatory, spatial-coverage and persistence limitations (18, 52–54). In MSLN-positive tumors with tumor-specific loss of the protective HLA allele, activator–blocker recognition provides a genotype-conditioned design rationale, but early clinical exposure and trafficking observations do not establish its superiority over alternative control strategies (28–32, 60). The proposed control choices and subsequent failure states are illustrative applications of the framework rather than validated treatment-selection rules.

Combination strategies should follow the same discipline. A second agent is justified when it closes a measured residual limitation and its contribution can be distinguished from that of the cell product. A separately dosed vascular or myeloid intervention may be preferable when reversibility is critical, whereas a cell-encoded output may be preferable when local delivery cannot reproduce the required spatial coupling. The relevant comparison is therefore among biologically plausible control locations rather than among technologies in isolation. **Table 4** applies this principle to three contrasting disease contexts, linking the initial control location to the residual limitation, first active counterfactual, falsifying pharmacodynamic measurement and likely mode of subsequent failure.

**Table 4:** Mechanism-resolving design across three contrasting disease contexts.

Decision element	CLDN18.2-positive gastric/GEJ	B7-H3, GD2 or IL-13Rα2 CNS	MSLN-positive, HLA-A*02-LOH	Refs
Initial control location	Prospective quantitative eligibility and systemic repeated infusion.	Locoregional or staged exposure with CSF monitoring.	Genotype-conditioned activator-blocker logic.	(18, 32, 52–54, 61, 62)
Residual limitation	Lesion-to-lesion target density, mucosal risk, peritoneal exposure and acquired target loss.	Spatial coverage, inflammation, antigen escape and functional persistence.	LOH heterogeneity, blocker saturation, tissue trafficking and untreated anatomical reservoirs, including CNS.	(18, 32, 52, 61)
First active counterfactual	Quantitative eligibility, affinity/density tuning or route optimization.	Route, schedule, cell state and rescue optimization before addition of multi-input logic.	Selection, affinity tuning or regional exposure compared with Tmod control.	(17, 22–25, 35, 36, 52, 63)
Mechanism-proximal PD and sampling	Baseline quantitative target-density mapping with serial assessment of tissue exposure, CAR abundance, mucosal injury and compartmental ctDNA where informative.	Baseline CSF and MRI; CSF at approximately 24–48 h, before redosing and during weeks 1–2 as an illustrative schedule. Measure compartmental CAR-T abundance and a prespecified activation readout; assess edema and target state. CSF exposure is not a direct measure of lesion engagement.	Baseline HLA genotype, tumor LOH and activating-antigen distribution, followed by peripheral/tumor CAR-T exposure and assessment of HLA-loss versus HLA-retaining compartments where feasible.	(18, 32, 52, 54, 61)
Finding that challenges the claim → adaptive action	Verified target state and exposure meeting the prespecified lesion criterion without the predicted target-linked tissue effect challenges that function claim; important mucosal injury challenges selectivity. Reassess eligibility, receptor threshold or exposure rather than increasing dose alone.	Verified target availability and prespecified, representative compartmental exposure, but no activation signal above the prespecified assay/baseline-variability threshold in at least two informative post-infusion assessments -> challenge the compartmental activation claim; investigate target state, cellular competence and spatial coverage before escalating dose. CSF alone does not establish intratumoral engagement.	Activity in HLA-retaining tissue despite verified activating-antigen and protective-HLA expression challenges the selectivity claim after competing causes of injury are assessed. Recheck LOH, activator/blocker balance and exposure. Tumor nonresponse alone cannot assign failure to the blocker.	(18, 28–32, 52–54, 61)
Handling inadequate exposure or non-informative sampling	A negative result from an informative representative tissue exposure assay suggests an exposure limitation; a poor-quality or unrepresentative biopsy remains non-informative. Neither alone falsifies target-dependent effector function.	An adequate but negative representative exposure assay suggests a delivery/persistence limitation. Mistimed, low-quality or anatomically unrepresentative CSF remains non-informative. Neither result establishes failure of intratumoral effector function. Repeat/alternative sampling only when clinically justified.	Insufficient tumor exposure cannot establish activator or blocker failure. Indeterminate LOH or unavailable informative HLA-retaining tissue prevents blocker-level attribution; use feasible orthogonal testing rather than infer function from genotype or receptor expression alone.	(18, 28–32, 52–54)
Likely migration after successful initial control	Antigen-low escape or ecosystem suppression.	Inflammation, spatial escape, antigen loss or exhaustion.	LOH escape or anatomical relocation, including CNS.	(32, 52, 56, 59)

A locoregional CNS program illustrates a bounded mechanistic claim: administration is followed by measurable activation in the sampled CNS compartment. An illustrative sampling plan could obtain baseline CSF and MRI, CSF approximately 24–48 h after the first infusion, a pre-redosing sample and a further sample during the first 1–2 weeks, with MRI at a protocol-defined time. These intervals are design examples rather than a validated universal schedule. Before enrollment, the protocol should define exposure and assay-quality criteria and a pharmacodynamic response threshold exceeding analytical and baseline biological variability. For this bounded claim, the expected finding is a post-infusion activation signal exceeding the prespecified threshold within an informative sampling window in which compartmental exposure meets the protocol-defined criterion. Such a result would support local activation but would not, by itself, establish tumor-cell killing or the independent contribution of route. Serial CSF CAR-T measurements and an IFNγ/CXCL9/CXCL10 readout are possible components of this assessment (18, 52–54). Repeated absence of the prespecified activation signal in at least two informative post-infusion samples, despite verified target availability and exposure meeting the prespecified compartmental criterion, would challenge this limited activation claim and prompt assessment of cellular competence, target state or spatial coverage rather than dose escalation alone. CSF CAR-T detection does not establish intratumoral target engagement, and CSF cytokines are not specific evidence of tumor-cell killing; a claim about lesion-level action requires a justified relationship to lesion exposure and additional target or tissue evidence. Corticosteroid use, infection and sampling timing must be considered before assigning mechanistic failure. Absent exposure in a representative, technically adequate sample is evidence of a possible delivery or persistence limitation, whereas a mistimed, low-quality or unrepresentative sample remains non-informative. Neither finding alone falsifies downstream effector function. Further sampling should use a clinically feasible alternative and should not proceed solely to satisfy a mechanistic endpoint when its risk is unjustified.

### 1.9 CMC and potency are part of mechanism resolution

A programmable cell product is interpretable only if its claimed mechanism can be measured before infusion and followed after administration. Conventional release testing establishes identity, viability, purity, sterility, vector-related attributes and a proximal activity measure. It does not necessarily establish a threshold, veto, temporal sequence or ecosystem-conversion claim. Product testing should therefore decompose the mechanism into input fidelity, decision fidelity, output potency, functional competence, off-condition safety and rescue performance (66, 67).

Routine release should use the smallest robust assay set sufficiently proximal to the mechanism for lot disposition. More physiologic organoids, mixed-cell systems, oxygen gradients, spatial matrices and serial stimulation may be better suited to characterization, comparability, stability and clinical development. The objective is not to force every complex model into release, but to justify a bridge from the release assay to the model supporting the clinical hypothesis.

Decision products require quantitative criteria. An AND system should specify minimum double-positive activity and maximum single-positive leakage. An inhibitory system should define veto performance across activating-input strengths. A density filter should define its transition band and sensitivity to receptor-expression drift. A hypoxia-regulated product should specify normoxic leakage, induction across oxygen gradients and decay after reoxygenation. An ecosystem-rewriting product should define the intended state change rather than only the amount of secreted payload.

External control must be developed in parallel. Drug-regulated or adaptor systems require exposure-response, tissue penetration, on-off and washout kinetics, immunogenicity and repeated cycling (69–74). Inducible caspase-9 can eliminate engineered cells rapidly but irreversibly and cannot retrieve mediators already released. Standard organ-specific rescue and cytokine-directed management remain necessary (79–82). These analytical requirements should remain explicitly connected to the biological decision that the product is intended to execute. **Table 3** therefore integrates the computational representation of each major control architecture with its transition-state assays, candidate potency measures, extended characterization, clinical pharmacodynamics, unsafe-state definition and available modes of control or rescue.

### 1.10 Roadmap for mechanism-resolving cellular pharmacology

Mechanism-resolving cellular pharmacology requires five linked commitments. Eligibility assays should measure the biological variable sensed by the product (83), including density, co-expression or genotype where relevant (84, 85). Each module expected to materially alter efficacy, safety or tissue exposure should be compared with a clinically credible lower-burden strategy addressing the same limitation (86, 87). Early trials should obtain the tissue or compartmental pharmacodynamics needed to determine whether the mechanism operated. Potency and rescue assays should reproduce the transition and unsafe states implicit in the clinical claim. Preclinical PSMA CAR-T engineering with a dominant-negative TGFβ receptor (88) and clinical evaluation of inducible-caspase-9-equipped GD2-CART01 in neuroblastoma (89) exemplify mechanistically distinct resistance and safety-control strategies. Cohort-to-cohort decisions should be prespecified so that human data revise eligibility, route, manufacturing state, circuit or combination rather than merely increase dose (90, 91).

Within-patient management and between-cohort redesign occur at different timescales. Clinicians can alter dose, schedule, route, adaptor exposure, drug-gated activity and rescue treatment after infusion. Most genomic features of an autologous product cannot be changed after administration (88); pharmacodynamic evidence instead informs the next cohort or product generation. **Figure 6** separates these feedback processes and applies the same endpoint to both: did the selected control operate in the intended tissue, did it relieve the specified limitation and which limiting state emerged next?

**Figure 6:**
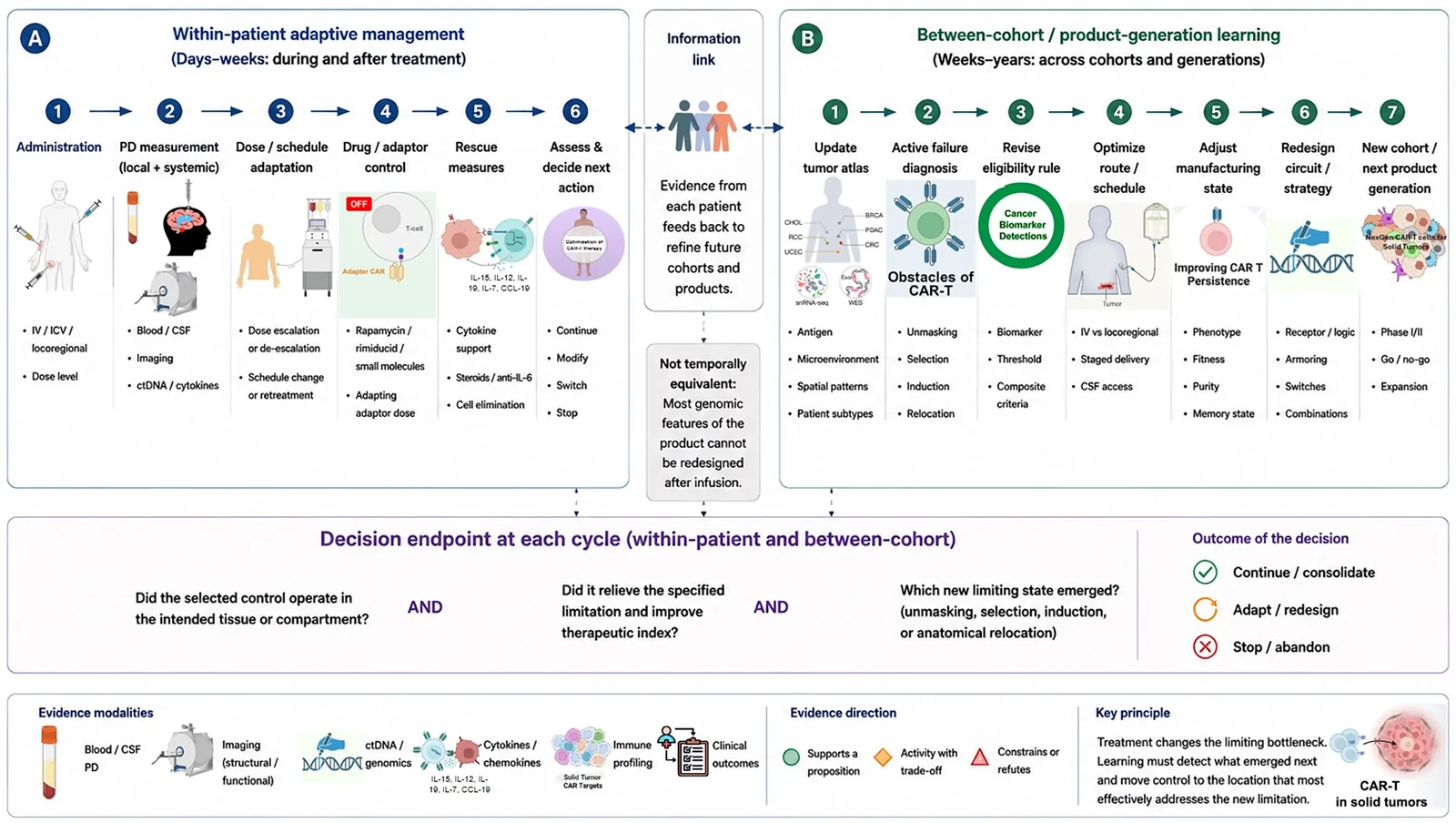
Mechanism-adaptive learning operates at two distinct timescales. Within-patient management includes administration, local and systemic pharmacodynamic measurement, dose or schedule change, drug/adaptor control and rescue. Between cohorts or product generations, the same evidence updates the tumor atlas, active failure diagnosis, eligibility rule, route, manufacturing state, circuit or combination strategy. These processes are information-linked but not temporally equivalent because most genomic features of an infused product cannot be redesigned after administration. Each cycle asks whether the selected control operated in the intended tissue, relieved the specified limitation and revealed an unmasked, selected, induced or relocated failure.

Several unresolved questions determine whether this approach can become practical. Antigen density and co-expression require standardization across immunohistochemistry, quantitative flow cytometry, spatial proteomics and functional assays. Environmental cues must be shown to discriminate tumors from infection, ischemia, fibrosis and wound repair. Tissue function may require local fluids, molecular imaging or perturbational biopsy, but sampling intensity must remain feasible for early trials. Mechanism-attribution rubrics will also require prospective inter-rater validation rather than retrospective expert placement alone.

The decisive comparison is no longer a simple CAR versus an elaborate CAR, but a comparison among alternative locations of control. When does a circuit outperform a quantitative diagnostic or affinity-tuned receptor? When does regional dosing outperform trafficking engineering? When does cell-state engineering outperform additional sensing? When does a separately dosed myeloid intervention provide safer and more reversible control than a permanent payload? These comparisons can identify intervention strategies that address a defined biological limitation while permitting assessment of their contribution to benefit, harm and treatment burden.

Mechanism-resolving development does not demand minimal engineering. It requires the engineered cell, enrollment-defining diagnostic, mechanism-proximal pharmacodynamic assays, route, schedule, analytical package, tissue-sampling plan and rescue strategy (89) to make one coherent and falsifiable claim. Complexity is justified when it resolves a demonstrated limitation better than a credible alternative and leaves evidence that the intended mechanism operated. Without that evidence, even clinical activity may not reveal how the next product should be designed.
